# Downregulation of Serum miR-133b and miR-206 Associate with Clinical Outcomes of Progression as Monitoring Biomarkers for Metastasis Colorectal Cancer Patients

**DOI:** 10.2174/0122115366266024240101075745

**Published:** 2024-01-12

**Authors:** Surasak Wanram, Namphon Klaewkla, Parichart Pinyosri

**Affiliations:** 1 College of Medicine and Public Health, Ubon Ratchathani University, Ubon Ratchathani, 34190, Thailand;; 2 Biomedical Science Research Unit, Ubon Ratchathani University, Ubon Ratchathani, 34190, Thailand

**Keywords:** Colorectal cancer, circulating microRNAs, prognostic biomarkers, serum miR-133b, serum miR-206, miRNAs validation

## Abstract

**Background:**

Colorectal cancer (CRC) is the third most common cancer in the world. Non-coding RNAs or microRNAs (miRNAs; miRs) biomarkers can play a role in cancer carcinogenesis and progression. Specific *KRAS* and *EGFR* mutation are associated with CRC development playing a role in controlling the cellular process as epigenetic events. Circulating serum miRs can serve for early diagnosis, monitoring, and prognosis of CRC as biomarkers but it is still unclear, clinically.

**Objective:**

To determine potential biomarkers of circulating serum miR-133b and miR-206 in CRC patients

**Methods:**

Bioinformatic prediction of microRNA was screened followed by TargetScanHuman7.2, miRTar2GO, miRDB, MiRanda, and DIANA-microT-CDS. Forty-four CRC serum (19 locally advanced, 23 distant advanced CRC) and 12 normal serum samples were subsequently extracted for RNA isolation, cDNA synthesis, and miR validation. The candidate circulating serum miR-133b and miR-206 were validated resulting in a relative expression *via* quantitative RT-PCR. Relative expression was normalized to the spike-internal control and compared to normal samples as 1 using the -2ΔΔCt method in principle.

**Results:**

Our results represented 9 miRs of miR-206, miR-155-5p, miR-143-3p, miR-193a-3p, miR-30a-5p, miR-30d-5p, miR-30e-5p, miR-543, miR-877-5p relate to *KRAS*-specific miRs, whereas, 9 miRs of miR-133b, miR-302a-3p, miR-302b-3p, miR-302d-3p, miR-302e, miR-520a-3p, miR-520b, miR-520c-3p and miR-7-5p relevance to *EGFR*-specific miRs by using the bioinformatic prediction tools. Our results showed a decreased expression level of circulating serum miR-133b as well as miR-206 associating with CRC patients (local and advanced metastasis) when compared to normal (*P* < 0.05), significantly.

**Conclusion:**

The circulating serum miR-133b and miR-206 can serve as significant biomarkers for monitoring the clinical outcome of progression with metastatic CRC patients. Increased drug-responsive CRC patients associated with crucial molecular intervention should be further explored, clinically.

## 1 INTRODUCTION

Colorectal cancer (CRC) affects over a quarter of a million people each year. The risk of developing CRC in developed countries is approximately 5% while the risk of developing adenoma, a precancerous cancer is 20%, approximately [1, 2]. When the disease is localized, effective treatment success rates range from 70-90% [3]. However, advanced CRC has a high mortality rate, consistently ranking as the third most common cause of cancer-related death worldwide [4]. Almost half of CRC patients are diagnosed at an early stage. If CRC has spread to adjacent tissues or organs and the regional lymph lymph nodes, the 5-year survival rate is close to 75%. If CRC has spread to distant areas of the body, the 5-year survival rate is only a quarter. The fact that too late diagnosis and various prognosis factors can affect the survival rate of CRC patients such as cancer stage, age, gender, histologic pattern, tumor grading, tumor size, and lymph node situation. Hence, early findings are important to decrease cancer mortality, and effective monitoring and screening assist in tracking the relapse of CRC patients [5]. Decision guidelines for CRC treatment are an effective way of decreasing cancer death. Molecular carcinogenesis of CRC is extremely complicated and diversified due to genetic and epigenetic instability during CRC development [6]. Accumulation of the genetic mutations and epigenetic alterations mechanism is very important to drive cancer progression through to tumor suppressor genes and oncogenes contributing manners [7]. Epidermal growth factor receptor (EGFR) is one of the most significant genes mediating CRC pathways. It is a transmembrane receptor human epidermal receptor (HER/ErbB) family receptor tyrosine kinase (RTKs). Controlling of the intracellular signal transduction affecting several pathways including rat sarcoma virus (RAS)/ mitogen-activated protein kinase (MAPK), phosphoinositide 3-kinase (PI3K)/Akt, signal transducer and activator of transcription (STAT) and SRC/FAK pathways, are revealed [8]. Kirsten RAS (KRAS) is a constituent of the EGFR signal transduction pathway and affects proliferation and angiogenesis [9]. The KRAS mutations are found in 40% of CRC development. Mutations of KRAS have reduced GTPase activity resulting in enhanced cell proliferation and related to early tumorigenesis affecting persistent growth fostering signals, potentially. The most common alterations found involve three codons that appear to play a key role in the progression of CRC [10]. The oncogenic EGFR and KRAS mutations are accountable for triggering the RAS/MAPK kinase pathway in CRC. It can promote cell division, migration, proliferation, apoptosis, angiogenesis, adhesion, and motility [11]. The Kirsten RAS (KRAS) is a component of the epidermal growth factor receptor (EGFR) signal transduction pathway, which affects proliferation, and angiogenesis. KRAS mutations are present in about 40% of CRC. KRAS mutations have reduced GTPase activity that results in increased cell proliferation potentially related to early tumorigenesis affecting more persistent growth-promoting signals. The most frequent alterations detected involve three codons that appear to play a major role in the progression of colorectal cancer [12]. MicroRNAs (miRNAs: miRs) are small non-coding RNA molecules that are 19-25 nucleotides in length. It plays a key role in post-transcriptional gene regulation in biological gene process and expression. Their involvements are important for not only proliferation and differentiation of normal cells to survive, but also contributing to tumorigenesis. The miRNAs are related to the development of several types of cancers such as brain tumors, lung cancer, breast cancer, liver cancer, prostate cancer, and CRC. Circulating miRNAs have also emerged as promising diagnostic biomarkers for CRC screening [13-15]. By the 3’UTR of target genes, miRNAs may be functionally driven as tumor suppressor genes or oncogenes by regulating different targets. The previous study showed that miR-18a [16], miR-155 [17], and miR-106b-5p [18] can inhibit proliferation, migration, invasion, and metastasis of CRC cells, whereas miR-433 [19], miR-885-5p [20] and miR-181a promote cell proliferation, migration, and invasion [21-23]. Moreover, no single miRNA alone has been identified as an ideal CRC biomarker up to now, a panel of miRNAs can be used to distinguish CRC patients from healthy controls with relatively high sensitivity and specificity, from testing in a large population of subjects. Interestingly, functional dysregulation of microRNA-1/133a and microRNA-206/133b clusters are important in human cancers [24]. Moreover, serum miR-133b and miR-206 are shown as potential novel diagnostic and prognostic biomarkers for CRC [25, 26]. The computational prediction *via* bioinformatic tools is one of the essential guidelines for the selection of miRNAs as the panel CRC biomarkers identify candidate miRNAs by using 3’UTR of KRAS and EGFR. So, the purpose of this study was to identify a potential candidate miRNA specific to KRAS and EGFR mutation in CRC using available bioinformatics tools for computational prediction. After that the circulating miRs that represent being as the candidate serum biomarkers were determined in quantitative real-time PCR.

## 2 MATERIALS AND METHODS

### 2.1 MiRNAs Prediction Databases

The ensemble database retrieved involves the 3′UTR sequence of *KRAS* (human *KRAS* 3′UTR based on transcript NM_033360 (chr12: 25205246-25250929)) and *EGFR* (human *EGFR* 3′UTR based on transcript NM_005228 (chr7: 55019017-55211628)) from NCBI database. The bioinformatic tools for miRNA prediction that relate to *KRAS* and *EGFR* were computed from TargetScanHuman7.2, miRTar2GO, miRDB, MiRanda, and DIANA-microT-CDS. The practical workflow was proven considering all miRNAs on target site predictions. The bioinformatics tools were used for predicting the result of the crucial computational tools including five distinct predictions. TargetScan software program was one of the most frequent user-friendly programs established on the two classic types for miRNA target prediction. It was a seed match sequence between miRNA and 3’UTR of a target gene using the base pairing rule and other features of this algorithm [27]. The prediction was ranked based on the predicted efficacy of targeting as assessed using cumulative weighted context++ scores of the sites that are free energy. MiRTar2GO was used to forecast miRNA target sites using relaxed miRNA with target binding characteristics. Performance of rank candidate interactions based on minimal free energy (MFE) of hybridization as a primary parameter. It is against other widely used miRNA target prediction algorithms resulting in miRTar2GO generating significantly higher F1 and G scores. MiRDB was an available online database for miRNA target prediction and functional annotation. Miranda was a resource for miRNA target predictions and miRNA expression. The principle of lower mirSVR score was a method for ranking microRNA target sites. The algorithm coached a regression model on the sequence and contextual features extracted from MiRanda-predicted target sites. The MiRanda-mirSVR was a competitive finding with other target prediction methods in classifying target genes and predicting the extent of their downregulation at the mRNA or protein levels. DIANA-microT-CDS was the 5^th^ version of the micro-T algorithm. It was instructed on a positive and negative set of miRNAs, specifically. The candidate miRNA prediction was employed according to the following selection criteria showing exhibited greater than or equal to 4 in 5 prediction tools.

### 2.2 Clinical Samples and Candidate miRNAs Validation

All subjects provided written informed consent with the approval of EC004/2021 according to ethical and legal standards. Forty-two serum of 19 CRC without metastasis (locally advanced) and 20 CRC with metastasis (23 distant advanced) were followed by TNM staging, recurrence, and metastases information. Twelve normal sera were compared as healthy control, simultaneously. Briefly, the serum was prepared using centrifugation of 3,500 rpm 10 min after 30 min at room temperature, and the serum was kept at -80 ◦C, immediately. Serum samples were isolated for total RNA using miRNeasy Serum/Plasma Kit (QIAGEN, Germany). Polyadenylation and reverse transcription for cDNA synthesis were performed using miRCURY LNA RT Kit, with UniSp6 RNA spike-in as a reverse transcription positive internal control (QIAGEN, Germany). Quantitative real-time RT-PCR was determined using miRCURY LNA SYBR Green PCR Kit (QIAGEN, Germany), with candidate miR-133b and 206 primers. in triplicate and carried out on the Rotor-Gene Q Platform (QIAGEN, Germany). Relative expression of each circulating miRNA was normalized to the spike-internal control and compared to normal samples as 1. The result was calculated using the -2ΔΔCt method in principle, finally.

## 3 STATISTICAL ANALYSIS

All statistical analyses were conducted by using IBM SPSS statistics base 22.0 for Windows. A *P*-value less than 0.05 was considered, significantly. The Mann-Whitney U test was conducted to compare two group of serum levels of miR-133b and miR-206.

## 4 RESULTS

### 4.1 Prediction of KRAS and EGFR-specific miRNAs

We found KRAS-specific miRs that were associated with 913 miRs, 63 miRs, 147 miRs, 330 miRs, 47 miRs, and 326 miRs with KRAS *via* TargetScanHuman7.2, miRTar2GO, miRDB, MiRanda, and Diana tools, respectively. Candidate miRs were selected according to established criteria. Our results found 9 miRs including miR-206, miR-143-3p, miR-155-5p, miR-193a-3p, miR-30a-5p, miR-30d-5p, miR-30e-5p, miR-543 and miR-877-5p. The candidate miR-206 was represented and selected for validation [25]. We found EGFR-specific miRs to 635 miRs, 36 miRs, 146 miRs, 166 miRs, 34 miRs, and 253 miRs as above. Candidate miRs were selected according to established criteria. Our prediction found 9 miRs including miR-133b, miR-302a-3p, miR-302b-3p, miR-302d-3p, miR-302e, miR-520a-3p, miR-520b, miR-520c-3p and miR-7-5p, respecttively. The candidate miR-133b was represented and selected for further validation [26] as shown in Fig. (**1**).

### 4.2 Downregulation of miR-133b and miR-206 in Serum CRC

Our results showed decreased relative expression of serum miR-133b and miR-206 in CRC patients when compared to healthy samples. The expression levels of serum miR-133b and miR-206 in CRC were determined by quantitative RT-PCR, resulting in serum miR-133b and miR-206 expression levels being reduced in CRC patients as shown in Figs. (**2** and **3**), respectively. Interestingly, the results showed a significant difference (*P* < 0.05) between CRC patients on clinical outcomes of progression with progressive local and advanced metastasis when compared to normal patients, respectively.

## 5 DISCUSSION

At the present time, there are many challenges and exciting relevance to circulating miRNAs and CRC. MiRNAs play a key regulatory role in CRC development. It may play an important role in the pathogenesis of CRC associated with the control progression of tumors by regulating the specific target genes. Therefore, the expression profiles of miRNAs as a prediction biomarker of the target genes in CRC [28]. Similarly, our results show the possibility of miRNAs associated with the gene targets, especially for KRAS and EGFR genes. The bioinformatic prediction of miRNAs relates to the KRAS gene indicating that miR-206, miR-143-3p, miR-155-5p, miR-193a-3p, miR-30a-5p, miR-30d-5p, miR-30e-5p, miR-543 and miR-877-5p, is significant. The miRNAs prediction associates with EGFR gene that show candidate miR-133b, miR-302a-3p, miR-302b-3p, miR-302d-3p, miR-302e, miR-520a-3p, miR-520b, miR-520c-3p, miR-7-5p and miR-875-5p. Nevertheless, there are complicated molecular interaction networks affecting the interactions of miRNAs and target mRNAs. That is why the prediction of miRNA targets is still a challenge and so important for computational analysis [29-32].

Previous studies represent that serum miRNAs are indicated as non-invasive biomarkers for various cancers, especially for colon cancer patients resulting in CRC screening, early detection, diagnosis, prognosis, and chemosensitivity [33-42]. The validation findings notice that decreased expression levels of candidate circulating serum miRNAs of miR-206 and miR-133b resulted in anti-onco-miRs as well as a prognostic biomarker associated with advanced CRC development [43-46]. Previous studies have shown that a unique role of miR-206 in 5-FU resistance in CRC is associated with reduced survival of colon cancer patients and supports the development of miR-206 mimic as a potential target for reversing drug resistance [47]. It is that serum miR-206 and miR-133b expression levels associated with clinical outcomes of progression in CRC, significantly. As previous studies report, miR-133b also acts as a function of the tumor suppressor gene and inhibits metastasis with cetuximab resistance in CRC patients [48, 49]. It may play a key role in characteristics of angiogenesis proliferation, and promotes apoptosis. In a study of lung cancer, miR-133b is shown that up-regulation of miR-133b reduces cell proliferation, induces cell apoptosis and G0/G1 phase arrest, and decreases cell invasion by inhibiting SOX9/b-catenin signaling [50]. It supports that serum miR-133b expression is markedly decreased in CRC and related to the aggressive progression of patients. These findings suggest that serum miR-133b might serve as a potential prognostic biomarker for CRC patients, clinically. The circulating serum miR-133b is a potential further prognostic biomarker for CRC patients, clinically [51-53]. The results present that miR-206 serves as the prognostic biomarkers of relevance to 5-FU resistance in CRC patients. Its regulation is associated with a reduction of CRC patients’ survival, supporting for development of miR-206 mimic as a potential target for reversing drug resistance [54]. Moreover, repression of miR-206 relates to cell proliferation, migration, and activated apoptosis [55]. Downregulation of miR-206 is closely associated with poorer prognosis outcomes of progression and shorter overall survival [56]. It could be suggested that miR-206 acts as a tumor suppressor gene, functionally. So, the circulating serum miR-133b and miR-206 have a great potential biomarker for serum diagnosis, monitoring, and prognosis of CRC patients [57-60].

Moreover, several previous studies have shown the downregulation of specific miRNAs in serum CRC patients compared to healthy individuals. These dysregulations suggest that these miRNAs may serve as potential diagnostic or prognostic biomarkers for CRC. Diagnostic biomarkers, certain miRNAs have shown consistent downregulation in serum CRC patients. For instance, miR-29a, miR-92a, and miR-143 are frequently reported to be decreased in CRC patients compared to normal. Detection of downregulation miRNAs in serum samples may help in early detection and diagnosis of CRC patients [61-63]. Prognostic biomarkers and the downregulation of specific miRNAs have also been associated with poor prognosis in CRC patients. For instance, reduced levels of miR-141, miR-143, and miR-378 have been linked to advanced-stage, lymph node metastasis, and overall survival. Importantly, these circulating miRNAs may serve as prognostic indicators for CRC patients and aid in treatment decisions [64-67]. Therefore, downregulated miRNAs in CRC often have crucial roles in tumor suppression, controlling key oncogenic pathways, and inhibiting metastasis [68, 48, 69-72]. By targeting specific genes involved in these pathways, miRNAs can regulate cell proliferation, apoptosis, angiogenesis, and epithelial-mesenchymal transition resulting in further specific target therapy [73-75]. However, it should be further designed on a larger cohort of CRC clinical cases, especially for diagnosis, prognosis, and monitoring treatments resulting in the clinical outcomes of metastatic CRC, efficiently.

## CONCLUSION

The downregulation of circulating serum miRNAs in CRC patients is very important in cancer research. MiRNAs are small non-coding RNA molecules that play crucial roles in the regulation of gene expression relevant to the epigenetic contributing factors. They have been implicated in various biological processes including of CRC development and progression. The association of specific circulating miRNAs in serum CRC patients and healthy individuals is applicable, significantly. It can be predicted and used for screening *via* available bioinformatic tools. Focusing on circulating serum miR-133b and miR-206 downregulation is associated with CRC severity and disease progression as well as metastasis. The epigenetic dysregulation suggests that circulating miRNAs may serve as the potential diagnostic or prognostic biomarkers of serum CRC patients, especially for monitoring treatments of metastatic CRC, clinically.

## Figures and Tables

**Fig. (1):**
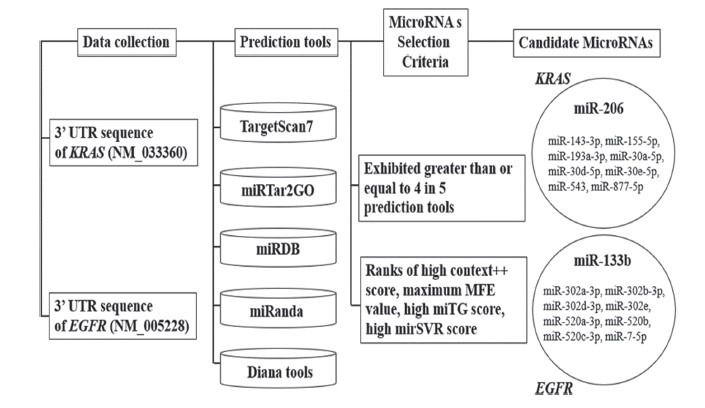
The computational bioinformatic prediction tools on interested target genes in colon cancer. A couple of interested EGFR and KRAS genes show significant candidate miRNA by using five prediction tools.

**Fig. (2):**
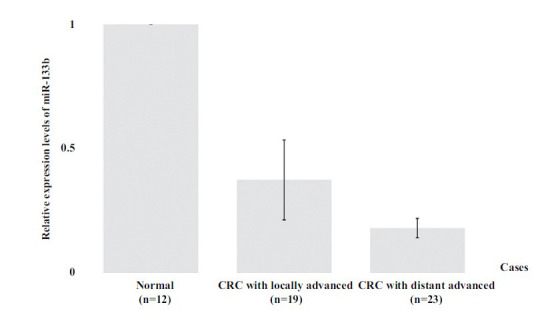
Relative expression levels of circulating miR-133b were significantly decreased in serum of locally advanced and distant advanced metastasis CRC patients, respectively, when compared to normal healthy.

**Fig. (3):**
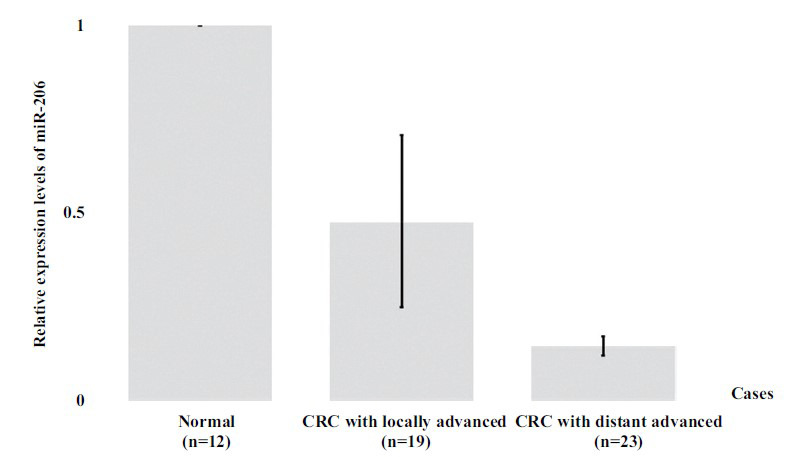
Relative expression levels of circulating miR-206 were significantly decreased in serum of locally advanced and distant advanced metastasis CRC patients, respectively, when compared to normal healthy.

## Data Availability

The data and supportive information is available within the article.

## LIST OF ABBREVIATIONS

CRC: Colorectal Cancer
KRAS: Kirsten RAS
MAPK: Mitogen-activated Protein Kinase
MFE: Minimal Free Energy
PI3K: Phosphoinositide 3-Kinase
RAS: Rat Sarcoma Virus
STAT: Signal Transducer and Activator of Transcription
